# The Emerging Role of *2OGDs* as Candidate Targets for Engineering Crops with Broad-Spectrum Disease Resistance

**DOI:** 10.3390/plants13081129

**Published:** 2024-04-17

**Authors:** Han Wang, Qinghe Chen, Wanzhen Feng

**Affiliations:** 1 School of Breeding and Multiplication, School of Tropical Agriculture and Forestry, Hainan University, Sanya 572025, China; hanwang@nwafu.edu.cn; 2 College of Plant Protection, China Agricultural University, Beijing 100193, China

**Keywords:** plant disease, broad-spectrum resistance, 2-oxoglutarate (2OG)-dependent oxygenases, crop improvement

## Abstract

Plant diseases caused by pathogens result in a marked decrease in crop yield and quality annually, greatly threatening food production and security worldwide. The creation and cultivation of disease-resistant cultivars is one of the most effective strategies to control plant diseases. Broad-spectrum resistance (BSR) is highly preferred by breeders because it confers plant resistance to diverse pathogen species or to multiple races or strains of one species. Recently, accumulating evidence has revealed the roles of 2-oxoglutarate (2OG)-dependent oxygenases (2OGDs) as essential regulators of plant disease resistance. Indeed, 2OGDs catalyze a large number of oxidative reactions, participating in the plant-specialized metabolism or biosynthesis of the major phytohormones and various secondary metabolites. Moreover, several *2OGD* genes are characterized as negative regulators of plant defense responses, and the disruption of these genes via genome editing tools leads to enhanced BSR against pathogens in crops. Here, the recent advances in the isolation and identification of defense-related *2OGD* genes in plants and their exploitation in crop improvement are comprehensively reviewed. Also, the strategies for the utilization of *2OGD* genes as targets for engineering BSR crops are discussed.

## 1. Introduction

Plant diseases caused by phytopathogens lead to substantial yield losses and a notable reduction in crop quality annually, becoming one of the important factors limiting agricultural production worldwide. The global crop losses due to pathogens and pests were estimated to be approximately 30.0% (24.6–40.9%) in rice, 22.5% (19.5–41.1%) in maize, 21.5% (10.1–28.1%) in wheat, 21.4% (11.0–32.4%) in soybean, and 17.2% (8.1–21.0%) in potato, respectively [1]. As a countermeasure, plants possess a two-layered immune surveillance system against a wide variety of invading microbes, including pattern-triggered immunity (PTI), activated through the recognition of highly conserved pathogen-associated molecular patterns (PAMPs) by cell surface-localized pattern-recognition receptors (PRRs), and effector-triggered immunity (ETI), initiated by intracellular resistance (R) proteins that directly or indirectly sense pathogen-encoded virulence effectors [2,3,4,5,6]. Although the activation mechanisms and the early signaling cascades of PTI and ETI are different, these two types of plant immunity converge on overlapping downstream cellular responses, including the induction of defense genes, the activation of mitogen-activated protein kinases (MAPKs), the accumulation of reactive oxygen species (ROS), and callose deposition [3,7].

Broad-spectrum resistance (BSR) is defined as plant disease resistance against at least two types of pathogen species or multiple members of one species [8]. Many genes involved in BSR have been identified and characterized in plants, such as those encoding membrane-associated PRRs, intracellular resistance proteins, defense-regulator proteins, pathogenesis-related (PR) proteins, susceptibility proteins, and nonhost resistance proteins [9]. For instance, FLAGELLIN SENSING 2 (FLS2), RLK-PRR EF-TU RECEPTOR (EFR), Xa21, lysin motif-containing proteins 4 (LYP4), LYP6, and elicitin response (ELR) function as PRRs to activate plant defense responses via sensing the matched PAMPs, and the ectopic expression of these genes in other plant species induces BSR to multiple pathogens [10,11,12,13,14,15,16,17,18,19,20,21,22,23,24]. Overexpressing the antimicrobial protein genes *CaAMP1* or *LjAMP2* in transgenic lines led to enhanced resistance against multiple fungal pathogens [25,26,27]. *TaPsIPK1*, encoding a receptor-like cytoplasmic protein kinase employed by the rust effector PsSpg1, has been found to be an important susceptibility gene in wheat, and the knockout of this gene confers BSR against *Puccinia striiformis* f. sp. *tritici* (*Pst*) races CYR32, CYR33, and CYR34 [28]. *NONHOST 1* (*NHO1*), *PENETRATION1* (*PEN1*), *PEN2*, and *PEN3* are four reported nonhost resistance genes that contribute to BSR against several host-nonspecific pathogens [29,30,31,32,33,34,35]. Additionally, quantitative trait loci (QTLs) conferring BSR have been cloned in different plants. Two QTLs, *Lr67*/*Yr46* and *Lr34*/*Yr18*/*Pm38*, were found to positively regulate BSR against various fungal pathogens that cause powdery mildew, stripe rust, and leaf rust in wheat [36,37,38,39,40]. The cucumber-resistant QTL *dm1*/*psl*/*cla*, a loss-of-susceptibility mutation of the *STAYGREEN* (*CsSGR*) gene, confers BSR to the obligate biotrophic oomycete *Pseudoperonospora cubensis*, the hemibiotrophic fungal *Colletotrichum orbicular*, and the bacterial *Pseudomonas syringae* pv. *Lachrymans* [41].

Moreover, 2-oxoglutarate (2OG)-dependent oxygenases (2OGDs) are soluble non-heme proteins distributed ubiquitously throughout the kingdoms of life, and they catalyze a variety of oxidative reactions, including hydroxylation, epimerization, demethylation, sequential oxidation, ring formation, ring expansion, cyclization, halogenation, epoxidation, and desaturation [42,43,44,45,46,47]. Commonly, 2OGDs utilize ferrous iron Fe(II) as a cofactor and employ 2OG and molecular oxygen (O_2_) as cosubstrates to catalyze the oxidation of a substrate, generating the desired product, carbon dioxide (CO_2_), and succinate. The 2OGD family can be categorized into three functionally distinct groups (designated as DOXA, DOXB, and DOXC) based on phylogenetic analyses [43]. The DOXA group includes the homologs of *Escherichia coli* AlkB, a DNA repair enzyme, and demethylates the alkylated DNA, RNA, and histones [48,49,50,51]. The DOXB group containing a prolyl 4-hydroxylase (P4Hc) domain functions as the key enzymes in the biosynthesis of collagen [52] and also catalyzes the post-translational modifications of the proline hydroxylation of plant cell wall proteins [53]. The DOXC group, the most functionally diverse and largest subfamily of plant *2OGD*s, participates in the specialized biosynthesis or metabolism of various phytohormones and secondary metabolites, such as gibberellins, auxin, ethylene, salicylic acid (SA), jasmonic acid (JA), flavonoids, tropane alkaloids, benzylisoquinoline alkaloids, coumarins, and glucosinolates, etc. [42,43,47]. 

In this review, the landmark discoveries of the roles of *2OGD* genes in plant immunity are revisited, and the recent advances regarding *2OGD*-mediated BSR are also summarized. Additionally, the molecular mechanisms of how *2OGD*s confer BSR to pathogens and the application of *2OGD*s in crop improvement for disease resistance are discussed.

## 2. The Discoveries of *2OGD*-Mediated Broad-Spectrum Disease Resistance

In 2005, eight *downy mildew-resistant* (*dmr*) mutants corresponding to six different loci were found to show reduced susceptibility to the downy mildew pathogen *Hyaloperonospora parasitica* via screening the ethyl methane sulfonate (EMS) mutants in the highly susceptible genetic background of the *Arabidopsis* line Ler *eds1-2* [54]. Among them, *DMR6* was then isolated via map-based cloning and characterized as *At5g24530*, which encodes an oxidoreductase belonging to the 2OG-Fe(II)-dependent oxygenase superfamily [55]. Intriguingly, the loss of function mutant *downy mildew-resistant 6* (*dmr6*) also exhibited elevated resistance against the oomycete *Phytophthora capsici* (*P. capsici*) and the bacterium *Pseudomonas syringae* (*P. syringae*), and overexpressing *DMR6* in *Arabidopsis* led to enhanced susceptibility to *Hyaloperonospora arabidopsidis* (*H. arabidopsidis*), *P. capsici*, and *P. syringae* [56]. Additionally, the overexpression of *DMR6-LIKE OXYGENASE 1* (*DLO1*) and *DMR6-LIKE OXYGENASE 2* (*DLO2*), two closely related homologues of *DMR6*, restored the susceptibility of the *dmr6* mutant to downy mildew *H. arabidopsidis*, similar to *DMR6* [56]. These important findings confirm that the *DMR6* and *DMR6* homologues serve as negative regulators of BSR in *Arabidopsis*, and represent the first example of *2OGD* functioning as a key component in BSR, which has attracted many colleagues to focus on advancing our understanding of this new field.

The knockdown of *StDMR6* via RNAi silencing and the knockout of *StDMR6-1* in potatoes resulted in enhanced resistance against the necrotrophic pathogen *Botrytis cinerea* (*B. cinerea*) and the hemibiotrophic pathogen *Phytophthora infestans* (*P. infestans*), respectively [57,58]. *Sldmr6-1* mutants exhibited increased disease resistance against three evolutionarily different classes of pathogens, including a fungus (*Pseudoidium neolycopersici*), an oomycete (*P. capsici*), and bacteria (*P. syringae* pv. *tomato*, *Xanthomonas gardneri*, and *Xanthomonas perforans*) [59]. The mutagenesis of *ObDMR6* confers resistance to the downy mildew pathogen *Peronospora belbahrii* in sweet basil, which is supported by the decreased pathogen biomass and sporangia production on the infected leaves [60]. The ectopic overexpression of *AhS5H1* or *AhS5H2*, the first susceptible gene characterized in peanut, blocked the induction of defense-related genes (*AtPR1* and *AtPR2*) upon chitin treatment and enhanced the susceptibility to *P. syringae* pv. *tomato* DC3000 in *Arabidopsis* [61]. The introduction of *Hv2OGO* into *dmr6* mutants fully restored the susceptibility of *Arabidopsis* to *Fusarium graminearum* [62]. The inactivation of *MusaDMR6* promoted the banana resistance against *Xanthomonas campestris* pv. *musacearum*, the causal agent of a devastating bacterial disease called Banana Xanthomonas wilt (BXW) [63]. In addition, *OsSAH2* and *OsSAH3* were recently reported to negatively regulate the rice resistance to *Magnaporthe oryzae* (*M. oryzae*), *Xanthomonas oryzae* pv. *Oryzae*, *Bipolaris oryzae*, and *Rhizoctonia solani* [64,65,66].

Apart from the *DMR6* and *DMR6* orthologs, other members of the DOXC subfamily have also been found to play essential roles in plant immunity. The transcripts of various *1*-*aminocyclopropane*-*1*-*carboxylic acid oxidase* (*ACO*) genes were induced by different pathogens in plants [67,68,69,70], suggesting the involvement of *ACOs* in the plant defense against pathogen infections. Silencing of *NbACO1* enhanced the host susceptibility of *Nicotiana benthamiana* to the hemibiotrophic fungi *Colletotrichum orbiculare*, with an accelerated switch from biotrophy to necrotrophy during the course of infection [67]. *TuACO3* encodes the 1-aminocyclopropane-1-carboxylic acid (ACC) oxidase and has been confirmed to positively regulate the wheat defense against the powdery mildew pathogen *Blumeria graminis* f. sp. *tritici* [70]. Four JA-induced *2OGD*s named *JASMONIC ACID OXIDASES* (*JAO1-4* or *JOX1-4*) act as important suppressors of *Arabidopsis* defenses against the fungal pathogen *B. cinerea* and the larvae of *Mamestra brassicae* [71,72]. *JAO2*-deficient lines display the constitutive expression of marker genes for the JA defense cascade and increased accumulation of multiple classes of antimicrobial compounds [72,73]. Furthermore, the resistance phenotype of *jao2* mutants can be recovered by the ectopic overexpression of *JAO2*, *JAO3*, or *JAO4* [72,73]. 

The information regarding the *2OGD* genes described above is summarized in Table 1.

## 3. The Roles of Phytohormones in Broad-Spectrum Disease Resistance

SA acts as an important player in the activation of plant innate immunity, including PTI, ETI, and systemic acquired resistance (SAR), which restricts the growth of invading pathogens [74,75]. The endogenous SA levels are demonstrated to be increased in plants upon stimulation with distinct pathogens, and SA-induction-insensitive mutants display compromised pathogen resistance [76,77,78,79,80,81,82,83,84,85]. The disruption of endogenous SA accumulation via ectopically expressing the bacterial salicylate hydroxylase gene, *NahG*, results in decreased resistance to the biotrophic and semi-biotrophic pathogens in transgenic tobacco and *Arabidopsis* plants [86,87]. Furthermore, the application of exogenous SA or SA analogs enhances the defense responses in plants against infectious pathogens [61,88,89,90,91]. Plants employ the primary metabolite chorismate as a precursor to synthesize SA via two different pathways with multiple steps, the isochorismate (IC) pathway and the phenylalanine ammonia-lyase (PAL) pathway [75,92]. Both the IC pathway and the PAL pathway contribute to the pathogen-induced SA accumulation and its functions in plant resistance because the inactivation mutants in the key components of these two pathways display lowered SA levels and enhanced susceptibility to many pathogens [78,79,84,85]. The alterations in the endogenous SA levels are sensed by the SA receptors (non-expressers of *PR* genes, NPRs), and the response signals are then transduced to activate the expression of the downstream *PR* genes, which encode anti-microbial proteins. In *Arabidopsis*, NPR1 is well-known to positively regulate the expression of downstream defense-related genes; however, NPR3 and NPR4 act as negative players in SA-mediated plant defense [93,94,95]. SA can be reversibly or irreversibly converted to bioactive derivatives via glycosylation, sulfonation, AA conjugation, methylation, and hydroxylation [75], thus helping plants to fine-tune SA homeostasis.

JA, the lipid-derived phytohormone, plays an essential role in the plant immunity against necrotrophic pathogens. The infection of these pathogens triggers the rapid accumulation of JA and jasmonoyl-L-isoleucine (JA-Ile), the major bioactive derivative of JA, which activate the downstream signaling to upregulate the production of the defense-related secondary metabolites and proteins in plants, such as alkaloids, phenylpropanoids, terpenoids, and several types of PR proteins [96,97]. JA is formed from 12-oxophytodienoic acid (OPDA) via an enzymatic reaction of 2-oxophytodienoic acid reductases (OPRs), and OPDA is synthesized from chloroplast membrane lipids by sequential catalytic reactions of lipoxygenases, allene oxide synthase, and allene oxide cyclase in different intracellular compartments [98,99]. JA can be further conjugated with isoleucine to synthesize JA-Ile via JA amido synthetase 1 (JAR1) in the cytosol [98,99]. The binding of JA-Ile with its co-receptor, the CORONATINE INSENSITIVE1 (COI1)/JASMONATE ZIM DOMAIN (JAZ) complex, leads to the degradation of JAZ through the 26S proteasome pathway, thereby releasing the suppression of the JAZ-interacting transcription factor MYC2 that activates various JA-responsive genes [98,99,100]. The JA biosynthetic and insensitive mutant plants displayed a remarkable reduction in resistance to necrotrophic pathogens. *Arabidopsis fad3*/*fad7*/*fad8* triple mutants were highly susceptible to the fungus *Pythium mastophorum*, and the exogenous application of methyl jasmonate could nearly restore the resistance against *P. mastophorum* [101]. In tomato, jasmonate-deficient mutants *def1* showed enhanced susceptibility to five pathogens, including two bacteria (*Xanthomonas campestris* and *P. syringae*), two fungi (*Fusarium oxysporum* f. sp. *lycopersici* and *Verticillium dahliae*), and an oomycete (*P. infestans*) [102]. Maize *opr7*/*opr8* double mutants lost the defensive ability of damping-off disease caused by the oomycete *Pythium aristosporum* [103]. The *coi1* mutants failed to activate the pathogen-induced expression of *PR*-*3*, *PR*-*4,* and *PDF1.2* in *Arabidopsis*, and exhibited elevated susceptibility to the fungal pathogens *B. cinerea* and *Alternaria brassicicola* but not *Peronospora parasitica* [104]. Although JA signaling was reported to function negatively regarding the defense against some hemibiotrophic bacteria in dicotyledonous plants [105,106], it also acts as a positive player in resistance against the hemibiotrophic pathogen *M. oryzae* in rice and viruses in both *Arabidopsis* and rice [107,108,109,110,111,112,113]. For instance, JA accumulation was triggered by the coat protein (CP) of *Rice stripe virus* (RSV), and then JAMYB, a transcription factor downstream of the JA signaling, upregulated the expression of *Argonaute 18* (*AGO18*), thereby activating the RNA silencing pathway for the rice antiviral defense [113].

The synthesis of ethylene starts from methionine (Met), and the amino acid is catalyzed to S-adenosyl-methionine (AdoMet) via the S-AdoMet synthase [114,115,116]. AdoMet is then catalyzed to 1-aminocyclopropane-1-carboxylic acid (ACC) through the 1-aminocyclopropane-1-carboxylate synthase (ACS), followed by the formation of ethylene from ACC via the ACC oxidase (ACO) [114,115,116]. Ethylene biosynthesis was induced by *M. oryzae*, and the increased ethylene production enhanced the rice blast disease resistance [68,117,118]. Furthermore, the inhibition of ethylene biosynthesis by aminoethoxyvinylglycine (AVG) decreased the rice resistance to blast [68,117,119]. Intriguingly, transgenic rice lines expressing *OsACS2* by a strong pathogen-induced promoter showed significant accumulation of ethylene upon *M. oryzae* infection and exhibited elevated resistance against rice blast and sheath blight [120]. A recent report found that PTI- and ETI-inducible OsMETS1, a methionine synthase deubiquitinated and stabilized by OsPICI1, promoted blast resistance via increasing the ethylene levels in rice [119]. Moreover, ethylene was confirmed to positively regulate the disease resistance against the hemibiotrophic and biotrophic pathogens in other plants, including soybean, tobacco, *A. thaliana*, and wheat [70,121,122,123]. Apart from ethylene accumulation, the components of ethylene signaling also contribute to plants’ defense against pathogens. For instance, overexpression of *ERF*, the ethylene responsive factor gene, elevated the host resistance to invading pathogens in many plants [124,125,126,127,128,129,130,131,132]. However, there are some cases showing that ethylene and ethylene signaling play negative regulatory roles in plant defense responses [133,134,135,136,137]. It seems that ethylene functions as a negative or positive regulator of disease resistance, relying on the pathogen type, plant species, and environmental conditions, which may be explained via the intricate mediation of the JA signaling and SA signaling through ethylene interaction [138,139]. 

## 4. Regulating Plant BSR with *2OGD*s via Altering the Levels of Phytohormones

**Figure 1:**
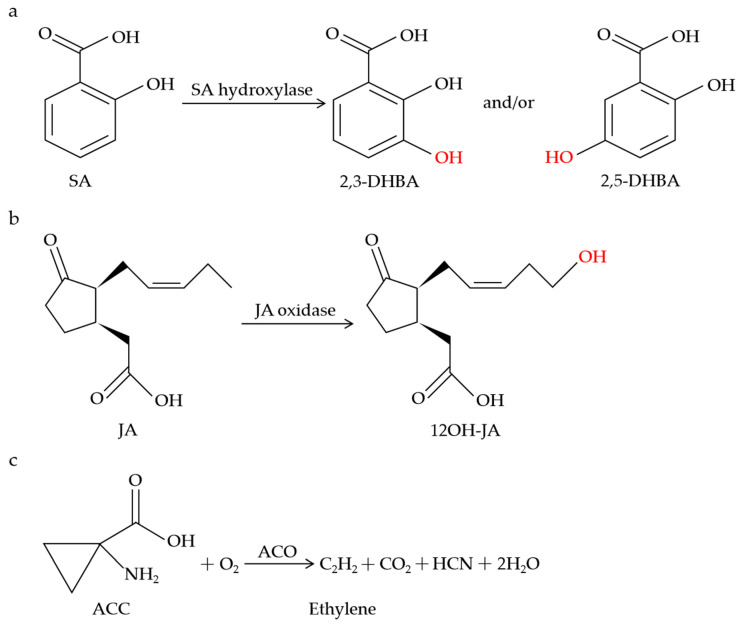
Schematic of metabolic conversions of SA, JA, and ethylene by 2OGDs. SA hydroxylase catalyzes SA to 2,3-DHBA and/or 2,5-DHBA (**a**). JA oxidase catalyzes the specific hydroxylation of JA to 12OH-JA (**b**). ACO catalyzes the biosynthesis of ethylene (**c**).

*The dmr6* mutants did not exhibit the constitutive expression of *PR*-*1*, and the pathogen growth was inhibited in the *dmr6* mutants without the accumulation of reactive oxygen intermediates (ROI) or obvious cell death [54], suggesting the important role of DMR6 in the cellular processes during pathogen infection. Subsequently, *dmr6*-mediated resistance was deduced from the increased expression of some defense-related genes or the altered levels of the DMR6 substrate and product [55]. It is worth mentioning that the SA levels in wild-type plants are approximately 10 times lower than in the *dmr6* mutant and more than 200 times lower than in the *dmr6*-*3*_*dlo1* double mutant [56]. Notably, DLO1 was identified and characterized as an SA 3-hydroxylase (S3H) that regulates leaf senescence via modulating the SA catabolism in *Arabidopsis* (Figure 1a) [140]. DLO1/S3H catalyzes SA to both 2,5-dihydroxybenzoic acid (2,5-DHBA) and 2,3-DHBA in vitro but only 2,3-DHBA *in planta* (Figure 1a) [140]. Given that *DMR6* functions redundantly with *DLO1* and the SA accumulation in the *dmr6* mutant is higher than that in the *dlo1* mutant, it is possible that DMR6 also catabolizes SA in a similar way to DLO1. As expected, DMR6 was discovered and functionally characterized as an SA 5-hydroxylase (S5H) via an enzymatic assay procedure (Figure 1a) [141]. DMR6/S5H catalyzes the specific hydroxylation of SA to 2,5-DHBA in vitro and *in planta*, with higher catalytic efficiency than DLO1/S3H [141].

JAO1-4 clustered between DLO1 (S3H) and amino-cyclopropane oxidase (ACCox), belonging to a four-member subclade named DOX 46 [43,72]. Based on this phylogenetic proximity, it was hypothesized that JAO1-4 could function in the oxygenation or hydroxylation reactions using hormone compounds as substrates. In support of this hypothesis, JAO2-4 specifically oxidized JA to 12OH-JA in vitro (Figure 1b), with no catalytic activity towards indole-acetic acid (IAA), SA, 12-oxophytodienoic acid (OPDA), or JA-Ile [72]. In *B. cinerea*-infected leaves, the accumulation of 12OH-JA was strongly decreased in the *jao2*, *jao3*, and *jao4* mutants relative to the WT, indicating that JAO2, JAO3, and JAO4 contribute to the production of 12OH-JA upon *B. cinerea* stimulation [72]. Furthermore, molecular genetic evidence showed that the constitutive expression of *ORA59*, *PDF1.2*, and *PR4* in the *jao2* mutants was strongly reduced or abolished by the inactivation of JAR1 or COI1, clearly demonstrating that the *jao2* defense phenotype relies on the generation and recognition of JA-Ile [72].

ACO catalyzes the final step of the ethylene biosynthetic pathway in which the ethylene precursor ACC is converted to ethylene and hydrogen cyanide (Figure 1c) [114,115,116]. It has been found that *ACO*-mediated resistance is associated with the formation of ethylene. Upon blast fungus (*Magnaporthe grisea*) infection, both the *OsACO7* mRNA transcripts and the ACO activity were highly increased, accompanied by elevated ethylene emissions, contributing to the resistance of young rice plants [68]. The rapid activation of *NtACO1* and *NtACO2* expression coincided with a dramatic increase in the ethylene levels and the activation of the NtMEK2-SIPK/WIPK cascade-mediated defense responses in tobacco plants infected by *Tobacco mosaic virus* (TMV) [142]. Also, a marked increase in the ACO activity and *ACO* gene expression was observed in tomato plants inoculated with *Xanthomonas campestris* pv. *vesicatoria* (XCV), similar to ethylene biosynthesis [143]. TuACO3 was reported to upregulate the production of ethylene to enhance the defense against powdery mildew in einkorn wheat [70]. *TuACO3*-silenced plants displayed reduced ethylene production and increased susceptibility to *Bgt*, whereas *TuACO3*-overexpressing transgenic lines exhibited elevated ethylene levels and enhanced resistance to *Bgt* in wheat [70]. Intriguingly, the expression of *TuACO3* was suppressed by a transcription factor, TuMYB46L, and the downregulation of *TuMYB46L* in wheat following *Bgt* infection led to increased expression of *TuACO3*, thus accumulating more ethylene to promote the einkorn wheat defense [70].

## 5. Conclusions and Perspectives

Plant diseases cause devastating yield losses in various crops annually and become a major threat to global food production. Different management practices have been adopted to control the diseases, among which the generation and cultivation of disease-resistant varieties is usually thought to be one of the most effective and promising strategies. In addition, durable and broad-spectrum disease resistance can be obtained via the inactivation of plant susceptibility (S) genes that are exploited by pathogens to facilitate successful infection and proliferation [144,145]. Recently, the rapid development of genome editing technologies has made it possible to precisely and efficiently mutate plant endogenous genes [146], which provides a foundation for creating disease-resistant plants via editing target genes. Taking advantage of this technology, the disease resistance was enhanced in many plants through the dysfunction of the S gene. For instance, *Mildew resistance locus O* (*Mlo*) is a well-known S gene to powdery mildew and is ubiquitously present across almost all higher monocots and dicots [147,148,149]. *Mlo* encodes a membrane-anchored protein with seven conserved transmembrane domains and functions in the processes of PM fungal penetration [150,151]. So far, researchers have successfully created PM-resistant tomato, grapevine, wheat, and soybean via the targeted mutagenesis of *Mlo* utilizing the CRISPR/Cas9 system [152,153,154,155,156].

**Figure 2:**
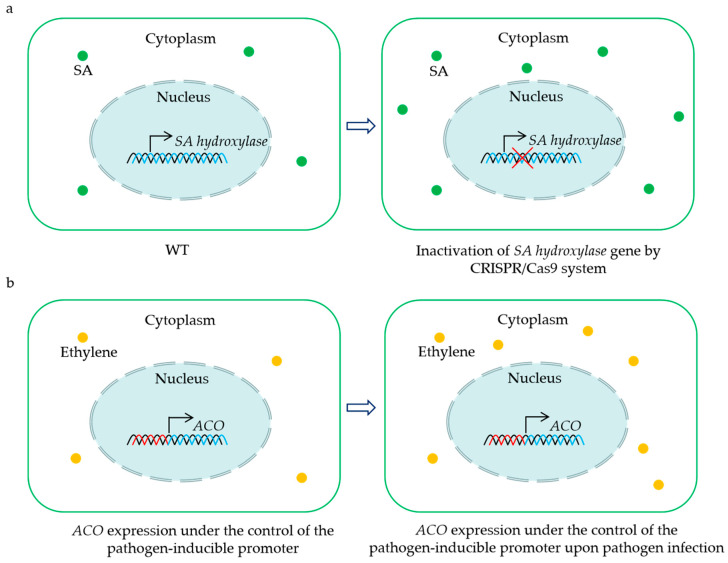
Representative strategies for the utilization of *2OGD* genes for creating crops with BSR. CRISPR/Cas9-mediated mutation of the *SA hydroxylase* gene results in sufficient SA accumulation to enhance disease resistance (**a**). Placing *ACO* gene under the control of the pathogen-inducible promoter leads to an induction of ethylene biosynthesis upon pathogen infection, and the increased levels of ethylene contribute to plant BSR (**b**). The green dots indicate salicylic acid (SA), and the yellow dots show ethylene.

As described in this paper, some *2OGD*s are S genes that negatively regulate the BSR in plants, including *JAO1-4* (*JOX1-4*), *DMR6*, and *DMR6* orthologs. This implies that these genes may be candidate targets for engineering crops with enhanced resistance to pathogens (Figure 2a). Until now, the CRISPR/Cas9-mediated mutation of a *DMR6* ortholog has conferred BSR in tomato and rice [59,64,65,66]. Intriguingly, the disruption of the *DMR6* ortholog does not influence the yield per plant of tomato and rice [59,65]. Moreover, elevated resistance to a single pathogen has been reported in banana, sweet basil, and potato via the inactivation of a *DMR6* ortholog by the CRISPR/Cas9 system [58,60,63]. It still needs to be evaluated whether these mutants exhibit enhanced resistance to other pathogens. Considering that the mutation of the *DMR6* ortholog may have potential negative effects on growth and yield, the CRISPR/Cas9-mediated knockdown of this gene represents another effective path for creating BSR crops without fitness costs. Recently, three amino acids in the substrate-binding sites were found to affect the enzyme activity of the DMR6 ortholog in rice, and substitutions of these amino acids led to a decrease in the oxidation of SA to 2,5-DHBA in vitro [64]. This suggests that base substitutions of the codes for key amino acids via base editors provide a possible strategy for enhancing the resistance of crops.

The overexpression of the key positive regulator of immunity in plants is also recognized as one of the strategies to engineer BSR crops. There are successful cases where *AtNPR1* overexpression has led to significantly enhanced BSR in diverse crop species without obvious alterations in their growth phenotype [157,158,159,160,161,162]. However, penalties in terms of plant development, yield, and virus resistance were also found in the transgenic strawberry or rice overexpressing *AtNPR1*, respectively [163,164,165]. Of note, expressing *AtNPR1*, which is controlled by the pathogen-induced promoter and the 5′ leader sequence, confers rice resistance to the bacterial leaf streak, blast, and blight in the field without fitness costs [166]. These findings establish an upgrade strategy for enhancing BSR in crops via the rapid and transient activation of plant defenses upon pathogen attacks. Based on this concept, we propose a possible method for engineering BSR crops by placing the *ACO* gene under the control of the pathogen-inducible promoter (Figure 2b). Although the positive role of *ACO* in BSR has been confirmed, this solution still needs to be further tested for controlling diseases and agricultural application under field conditions.

The *2OGD* superfamily comprises a large number of members and ranks second in the plant enzyme family [43]. Generally, *2OGD*s function in plants’ primary or secondary metabolism, which plays an essential role in plant defenses. However, there are only a few *2OGD* genes that are demonstrated to act as important players in plant immunity. To enrich our knowledge regarding the roles of *2OGD* genes in plant disease resistance, more research on the isolation and characterization of these genes is needed in the future. Moreover, understanding the detailed molecular mechanism of how *2OGD* genes contribute to the plant immune response may provide novel insights for engineering BSR crops. Additionally, the rapid development and application of CRISPR/Cas9-mediated genome editing technology in different crops will accelerate the use of *2OGD* genes to create BSR crops.

## Figures and Tables

**Table 1:** *2OGD* genes involved in plant disease resistance.

Plant	Gene Name	Protein Function	Contribution to Plant Immunity	Pathogen	Reference
*Arabidopsis*	*AtDMR6*	Hydroxylating SA	Negative	*Hyaloperonospora parasitica*	[54,56]
				*Phytophthora capsici*	
				*Pseudomonas syringae*	
				*Hyaloperonospora arabidopsidis*	
Potato	*StDMR6*	Hydroxylating SA	Negative	*Botrytis cinerea*	[57,58]
				*Phytophthora infestans*	
Tomato	*SlDMR6-1*	Hydroxylating SA	Negative	*Pseudoidium neolycopersici*	[59]
				*Phytophthora capsici*	
				*Pseudomonas syringae* pv. *tomato*	
				*Xanthomonas gardneri*	
				*Xanthomonas perforans*	
Sweet basil	*ObDMR6*	Hydroxylating SA	Negative	*Peronospora belbahrii*	[60]
Peanut	*AhS5H1*	Hydroxylating SA	Negative	*Pseudomonas syringae* pv. *tomato* DC3000	[61]
Peanut	*AhS5H2*	Hydroxylating SA	Negative	*Pseudomonas syringae* pv. *tomato* DC3000	[61]
Barley	*Hv2OGO*	Hydroxylating SA	Negative	*Fusarium graminearum*	[62]
Banana	*MusaDMR6*	Hydroxylating SA	Negative	*Xanthomonas campestris* pv. *musacearum*	[63]
Rice	*OsSAH2*	Hydroxylating SA	Negative	*Magnaporthe oryzae*	[64,65,66]
				*Xanthomonas oryzae* pv. *oryzae*	
				*Bipolaris oryzae*	
				*Rhizoctonia solani*	
Rice	*OsSAH3*	Hydroxylating SA	Negative	*Magnaporthe oryzae*	[64,65,66]
				*Xanthomonas oryzae* pv. *oryzae*	
				*Bipolaris oryzae*	
				*Rhizoctonia solani*	
Tobacco	*NbACO1*	Biosynthesizing ethylene	Positive	*Colletotrichum orbiculare*	[67]
Wheat	*TuACO3*	Biosynthesizing ethylene	Positive	*Blumeria graminis* f. sp. *tritici*	[70]
*Arabidopsis*	*AtJAO1*	Hydroxylating JA	Negative	*Botrytis cinerea*	[71,72]
*Arabidopsis*	*AtJAO2*	Hydroxylating JA	Negative	*Botrytis cinerea*	[71,72]
*Arabidopsis*	*AtJAO3*	Hydroxylating JA	Negative	*Botrytis cinerea*	[71,72]
*Arabidopsis*	*AtJAO4*	Hydroxylating JA	Negative	*Botrytis cinerea*	[71,72]

## Data Availability

Table 1 and Figure 1 and Figure 2 are available upon request from H.W. and W.F.
